# Greenland Ice Sheet Surfaces Colonized by Microbial Communities Emit Volatile Organic Compounds

**DOI:** 10.3389/fmicb.2022.886293

**Published:** 2022-06-07

**Authors:** Eva L. Doting, Cleo L. Davie-Martin, Anders Johansen, Liane G. Benning, Martyn Tranter, Riikka Rinnan, Alexandre M. Anesio

**Affiliations:** ^1^Department of Environmental Science, iClimate, Aarhus University, Roskilde, Denmark; ^2^Terrestrial Ecology Section, Department of Biology, University of Copenhagen, Copenhagen, Denmark; ^3^Interface Geochemistry, German Research Centre for Geosciences, GFZ Potsdam, Potsdam, Germany; ^4^Department of Earth Sciences, Freie Universität Berlin, Berlin, Germany

**Keywords:** VOC, glacier ice algae, ice melt, algal bloom, cryoconite holes, fungi, biogenic volatiles, red snow

## Abstract

Volatile organic compounds (VOCs) are emitted by organisms for a range of physiological and ecological reasons. They play an important role in biosphere–atmosphere interactions and contribute to the formation of atmospheric secondary aerosols. The Greenland ice sheet is home to a variety of microbial communities, including highly abundant glacier ice algae, yet nothing is known about the VOCs emitted by glacial communities. For the first time, we present VOC emissions from supraglacial habitats colonized by active microbial communities on the southern Greenland ice sheet during July 2020. Emissions of C5–C30 compounds from bare ice, cryoconite holes, and red snow were collected using a push–pull chamber active sampling system. A total of 92 compounds were detected, yielding mean total VOC emission rates of 3.97 ± 0.70 μg m^–2^ h^–1^ from bare ice surfaces (*n* = 31), 1.63 ± 0.13 μg m^–2^ h^–1^ from cryoconite holes (*n* = 4), and 0.92 ± 0.08 μg m^–2^ h^–1^ from red snow (*n* = 2). No correlations were found between VOC emissions and ice surface algal counts, but a weak positive correlation (*r* = 0.43, *p* = 0.015, *n* = 31) between VOC emission rates from bare ice surfaces and incoming shortwave radiation was found. We propose that this may be due to the stress that high solar irradiance causes in bare ice microbial communities. Acetophenone, benzaldehyde, and phenylmaleic anhydride, all of which have reported antifungal activity, accounted for 51.1 ± 11.7% of emissions from bare ice surfaces, indicating a potential defense strategy against fungal infections. Greenland ice sheet microbial habitats are, hence, potential sources of VOCs that may play a role in supraglacial microbial interactions, as well as local atmospheric chemistry, and merit future research efforts.

## Introduction

Volatile organic compounds (VOCs) are emitted by plants and microorganisms (Peñuelas and Llusià, 2004; Schmidt et al., 2015) for a variety of physiological and ecological reasons. They are a highly reactive part of the carbon cycle and are relevant to atmospheric chemistry because they modify the oxidation capacity of the atmosphere. For example, the presence of VOCs promotes the formation of tropospheric ozone (Atkinson, 2000) and contributes to the formation of secondary organic aerosols (Laothawornkitkul et al., 2009), which can serve as cloud condensation nuclei and affect cloud formation (Tsigaridis and Kanakidou, 2007). VOC emissions from many types of vegetation, such as trees and other plants, have been extensively studied (Peñuelas and Llusià, 2001, 2003; Rap et al., 2018) and their regional, as well as global, estimates are important parameters in atmospheric chemistry models that are used to study the global climate (Guenther et al., 2012). In contrast, VOC emissions from the world’s ice sheets have received much less attention, as they are devoid of trees and higher plants.

Arctic and subarctic vegetation in non-glaciated terrain has been shown to emit VOCs, and their emission rates are affected by climate warming (Faubert et al., 2010; Kramshøj et al., 2016). In addition, VOCs are emitted by thawing permafrost soils (Kramshøj et al., 2018), subarctic peatland, and lakes (Seco et al., 2020), as well as marine phytoplankton and algae associated with sea ice (Granfors et al., 2013). Snow was previously considered to merely be a sink for aerosols, but it is now known to be highly photochemically active (Sumner and Shepson, 1999) and plays a role in the production and release of a range of trace gases into the atmosphere (Grannas et al., 2004, 2007).

Until recently, the cryosphere was thought to be too cold and too devoid of life to present a significant source of VOCs. Ice sheets and glaciers are part of the cryospheric biome (Anesio and Laybourn-Parry, 2012) and are inhabited by active microbial communities consisting of algae, protozoa, bacteria, fungi, and viruses. These microorganisms both survive and thrive in the extreme environments typical of the cryosphere, utilizing a range of cold adaptations related to their cell metabolism (Cavicchioli et al., 2002). During the melt season, liquid water provides habitats for microbial communities that show activity comparable to that of soils and sediments at temperate latitudes (Anesio et al., 2009; Hodson et al., 2010). Algal blooms darken the ice surface due to pigments associated with the glacier ice algae *Ancylonema alaskanum* [previously *Mesotaenium berggrenii* (Procházková et al., 2021)] and *Ancylonema nordenskiöldii*, significantly reducing its albedo (Yallop, 2012; Lutz et al., 2018; Williamson et al., 2020). Bacteria, fungi, and viruses are also associated with ice surface algal blooms (Bellas et al., 2015; Nicholes et al., 2019; Perini et al., 2019).

Additionally, localized aggregates of dark organic-rich particles melt into the ice and form small melt ponds, known as cryoconite holes, which cover between 1 and 10% of the ablation zone. A different community of bacteria, viruses, algae, and cyanobacteria colonizes these aggregates, which accumulate at the bottom of the holes, and plays an important role in biogeochemical cycling in supraglacial systems (Anesio et al., 2009; Cameron et al., 2012). Finally, snow can host dense populations of phytoflagellates, such as *Chlamydomonas* cf. *nivalis* (partly referred to as *Sanguina nivaloides*; Procházková et al., 2019), *Raphidonema nivale*, and *Chloromonas nivalis*, which are responsible for the “red snow” phenomenon (Remias, 2012). Red snow algae present a potential sink of CO_2_ (Williams et al., 2003) and play a role in decreasing snow albedo (Lutz et al., 2016). While microbes are known to emit a range of VOCs (Schmidt et al., 2015; Weisskopf et al., 2021), to the best of our knowledge, no studies have assessed the *in situ* emission of VOCs from microbial communities inhabiting the snow and ice surfaces of land-based ice masses.

In this study, we report the first estimates of VOC emission rates from Greenland ice sheet surfaces colonized by microbial communities. We hypothesize that supraglacial microbes emit VOCs that may represent an important local source of volatiles in a region with few other sources of VOCs.

## Materials and Methods

### Site Description

**FIGURE 1:**
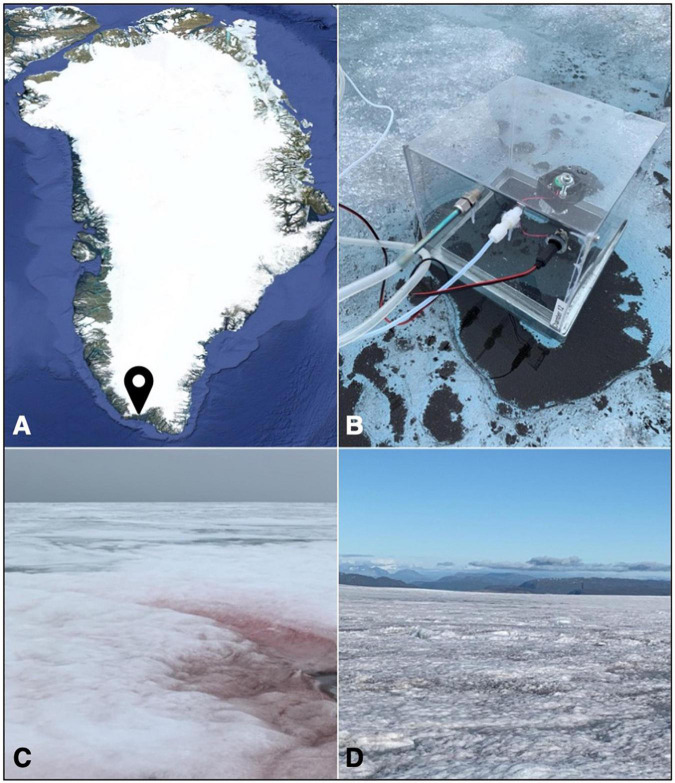
**(A)** Map of Greenland showing the location of the 2020 field site; **(B)** the sampling setup used in this study, with the sorbent tube for sample collection (left), the air inlet (middle) and the power supply for the internal fan (right), and the chamber base placed in a large cryoconite hole; **(C)** a red snow patch at the start of the sampling campaign; and **(D)** bare ice surface colonized by algal blooms.

VOC emissions were measured near the 2020 Deep Purple^1^ basecamp (∼ 61° 05′ N, 46° 50′ W), which was established on the southern Greenland ice sheet (Figure 1A), between 3 July and 18 July 2020, to study a variety of processes related to biological darkening of the Greenland ice sheet. VOC sampling sites were located approximately 700 m from the PROMICE^2^ (Programme for Monitoring of the Greenland Ice Sheet) weather station QAS_M, from which we retrieved hourly data for atmospheric temperature, relative humidity, and incoming shortwave radiation (W m^–2^) during sample collection. Melting of the snowpack was at an advanced stage during early July, with only a thin snow cover remaining over some areas. The southern Greenland ice sheet is experiencing increasing rainfall (Niwano et al., 2021) and the 2020 basecamp area received precipitation in the form of rain on 7 days of the campaign period. At the basecamp site, the average air temperature was 3.3 °C, the average incoming shortwave radiation was 247 W m^–2^, and the average number of daylight hours was 18.6.

### Sampling of Volatile Organic Compounds

VOC emissions from red snow habitats (Figure 1C) were collected on 3 July and 5 July, after which VOC emissions from the only remaining snow patches that covered crevasses and moulins could not be collected safely. In contrast, microbial colonization of the bare ice surface became more widely distributed as the melt season progressed. VOC emissions from three distinct habitats, namely, “dark ice” (bare ice surfaces with visible particles or algal bloom, Figure 1D), “clean ice” (bare ice surfaces without macroscopically visible particles or algal bloom), and cryoconite holes (Figure 1B), were collected between 6 July 2020 and 15 July 2020.

VOC emissions were collected using a push–pull enclosure setup (Tholl et al., 2006; Valolahti et al., 2015) with transparent polycarbonate chambers (thickness 1.5 mm, 220 mm × 220 mm, height 200 mm; Vink Finland, Kerava, Finland). Metal chamber bases were placed on the sampling sites [red snow (*n* = 2), bare ice (*n* = 31), or cryoconite holes (*n* = 4)] and left to melt into the ice for approximately half an hour. In the case of cryoconite holes, larger holes were selected so that the chamber bases could be placed within the water column to avoid collection of emissions from the surrounding bare ice surface. The grooves in the chamber bases were filled with water before placing the chamber enclosures over the bases, creating an airtight seal. Chambers were flushed at a flow rate of 1,000 mL min^–1^ for 10 min using battery-operated pumps connected *via* Teflon tubing. Incoming air was purified during flow through a PTFE filter and an activated charcoal filter to remove particles and VOCs, as well as by flow through a potassium iodide ozone scrubber (Ortega and Helmig, 2008). Following the 10-min flushing, a stainless steel sorbent tube containing 150 mg Tenax TA and 200 mg Carbograph 1TD (Markes International, C2-AXXX-5032) was inserted between the chamber and the outflow line. The outflow rate was set to 200 mL min^–1^ for the duration of the sample collection (between 0.5 and 5.5 h), while the inflow rate was set to 210 mL min^–1^ to maintain a slight overpressure in the chamber and prevent ambient air from entering the chamber. The chambers were equipped with a fan to ensure a well-mixed headspace. Temperature and relative humidity in the chamber headspace were logged once per minute using a shaded iButton sensor (Hygrochron DS 1923-F5, Maxim Integrated Products Inc., CA, United States).

After sample collection, cartridges were sealed with Teflon-coated brass caps and stored refrigerated, when possible, until analysis in Denmark. The chambers were removed, and, for bare ice samples, the top 2 cm of the ice within the chamber base was scraped off with a preconditioned ice axe and collected in sterile Whirl-Pak bags. Once melted, samples were homogenized and 2 mL of each sample was transferred to a 2-mL Eppendorf tube. The samples were fixed with glutaraldehyde (2% final concentration) and stored in the dark at 4 °C until counting. Microscope counts of glacier ice algae and snow algae were performed on a Fuchs-Rosenthal hemocytometer (3.2 μL, Lancing, United Kingdom) using an inverted light microscope (Olympus CK2). A minimum of 100 cells were counted where possible, but counts of the lowest cell concentrations contained fewer cells.

Procedural blank VOC collections were performed in the laboratory in Denmark to account for VOCs released from the sampling materials and analytical systems. The sampling setup (chamber base and chamber enclosure, with fan and iButton sensor) was placed on top of a VOC-free polyethylene terephthalate film (prebaked at 120 °C for 1 h) in a refrigerated chamber (0 °C) that was equipped with adjustable lights (Valoya C65 with NS12 spectrum, Helsinki, Finland) to mimic natural sunlight. Blanks were collected for 1 (*n* = 6), 2 (*n* = 2), and 4 (*n* = 2) h to account for potential background compounds, as well as compounds that were released continuously over time, using VOC-free air (Parker ChromGas Zero Air Generator) for inflow. The limitations of the sampling setup used in this study, such as the lack of steady state in the beginning of the sampling period and the possibility of compounds adhering to the chamber walls, have previously been discussed by Faubert et al. (2012).

### Analysis of Volatile Organic Compounds

Samples were analyzed by gas chromatography–mass spectrometry (GC–MS) (7890A Series GC coupled with a 5975C inert MSD/DS Performance Turbo EI System, Agilent Technologies, Santa Clara, CA, United States). Sorbent tubes were thermally desorbed (TD-100xr, Markes International, Llantrisant, United Kingdom) at 250 °C for 10 min, cryo-focused at −5 °C, and injected onto an HP-5 capillary column (length 50 m, diameter 0.2 mm, and film thickness 0.33 mm) with helium as carrier gas (1.2 mL min^–1^). During analysis, the column was kept at 40 °C for 3 min, then raised to 210 °C at a rate of 5 °C min^–1^, and finally to 250 °C at a rate of 20 °C min^–1^. Duplicate standard solutions (Supplementary Table 1) were injected into sorbent tubes and analyzed alongside samples at the start and end of each sample batch.

Chromatograms were analyzed using PARADISe (version 3.88) software (Johnsen et al., 2017). A total of 92 compounds were identified using pure standards where available (Supplementary Table 1), or were tentatively identified using the NIST Mass Spectral Library (version 2.2, 2014). Tentative identities were assigned if the NIST match factor (MF) was higher than 800 (the NIST Mass Spectrometry Data Center refers to MFs >800 as a good match) and the probability was higher than 30%. A tentative compound class was assigned (e.g., unknown alkane 1) for database hits that did not meet these conditions, but where at least two of the top three NIST matches had the same molecular formula. Compounds were classified as “oxygenated VOC” (e.g., unknown OVOC 1) when the top three NIST matches suggested different molecular formulae but all formulae contained oxygen, or as “other VOC” (e.g., unknown other 1) if they did not. Compounds were grouped into one of the following classes: alkanes, alkenes, terpenoids, oxygenated benzenoids, oxygenated VOCs, nitrogen-containing VOCs, and other VOCs. Concentrations were obtained by comparing peak areas between samples and standards, using the known mass of the standard injected. The closest structurally related standard (Supplementary Table 1) was used for quantification purposes for compounds that did not have an exact match with any of the standards injected.

Emission rates were calculated according to Faubert et al. (2012). In short, the VOC mass in the sorbent tube was divided by the sampled volume (a function of inflow rate and sampling time) to obtain the VOC concentrations in μg L^–1^ air. Average concentrations in the procedural blanks were subtracted from those detected in the samples, taking into account the sampling time for compounds that increased over time in the blank measurements. Compounds for which sample concentrations were less than two times higher than concentrations detected in the blanks were excluded from the dataset. After blank corrections, only the compounds with concentrations above zero in at least 50% of the samples for each habitat were included in the final analysis. The VOC concentrations were then divided by the sampling mid-time, multiplied by the total chamber volume, and normalized to the plot surface area to obtain emissions rates in μg m^–2^ h^–1^. All analyses were performed in R version 3.6.3 (R Core Team, 2015). Correlation analyses were performed using the Pearson correlation. Data on individual VOC emission rates were log(*x* + 1) transformed and unit variance scaled prior to principal component analysis (PCA).

## Results

### Sampling Conditions

Weather conditions during the sampling campaign were highly variable, with bad weather conditions making sample collection impossible on 7 out of 16 possible sampling days. This was because of the risk of water damage to the electronics in the sampling system during periods of heavy rain, or persistent cloud cover causing a lack of charge on the solar panels that supplied the camp, making it impossible to charge the battery-operated pumps. Samples were collected whenever possible between 10 AM and 8.30 PM, covering most of the natural variation in the environmental conditions at the sampling site (Table 1). The temperature and relative humidity inside the sampling chambers during a single sample collection varied by, on average, 2.3 °C and 6.3%, respectively. The average temperature inside the sampling chambers did not correlate with the air temperature during sample collection (*r* = −0.15, *p* = 0.50, *n* = 23), but correlated positively with the average incoming shortwave radiation at the time of sample collection (*r* = 0.63, *p* = 0.0012, *n* = 23).

**TABLE 1:** Summary of sampling conditions.

Name	Bare ice	Cryoconite hole	Red snow
Number of samples	31	4	2
Sampling duration (h)	0.5–5.6	2–4.2	2–5.3
Air temperature (°C)	1.7–4.5	2.2–4.0	2.2–4.6
Average chamber temperature (°C)	2.9–9.5	3.1–7.7	n.a.
Air RH (%)	77–99	83–99	66–89
Average chamber RH (%)	63–89	70–91	n.a.
Average incoming shortwave radiation (W m^–2^)	216–824	216–550	550–823
Glacier ice algae (cells mL^–1^)	0–94,000	n.a.	n.a.
Snow algae (cells mL^–1^)	0–1,500	n.a.	n.a.

*n.a., not available. Air temperature and relative humidity data were obtained from the PROMICE automatic weather station QAS_M, with sensors located approximately 2.6 m above the bare ice surface.*

### Volatile Organic Compound Emissions

**FIGURE 2:**
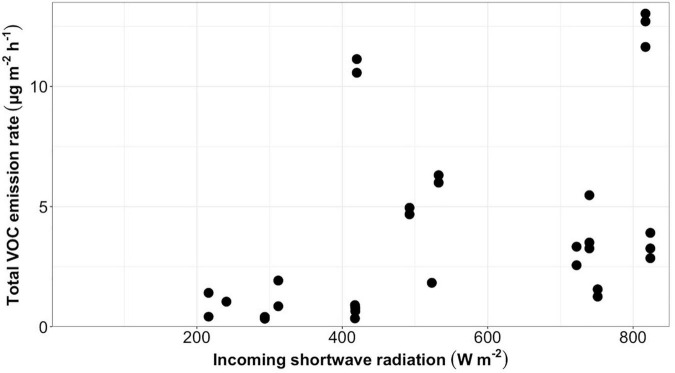
Total VOC emission rates (μg m^–2^ h^–1^) from bare ice surfaces (*n* = 31) plotted against average incoming shortwave radiation (W m^–2^) during sample collection.

Total VOC emission rates from bare ice had a low but statistically significant positive correlation (*r* = 0.43, *p* = 0.015, *n* = 31) with the incoming shortwave radiation averaged over the sample collection period for each sample (Figure 2). A similar significant positive correlation between emission rates and incoming shortwave radiation averaged over the sample collection time was found for all compound classes into which detected VOCs were grouped (Table 2). Total VOC emission rates from bare ice did not correlate significantly with chamber temperature (*r* = 0.37, *p* = 0.084, *n* = 23).

**TABLE 2:** Pearson’s correlation coefficients between emission rates (μg m^–2^ h^–1^) from bare ice surfaces (*n* = 31) per compound class and incoming shortwave radiation averaged during sample collection.

	Average incoming shortwave radiation (W m^–2^)
Alkanes	0.40*
Alkenes	0.47**
Terpenoids	0.35*
Oxygenated benzenoids	0.39*
Other oxygenated VOCs	0.39*
Nitrogen containing VOCs	0.42*
Other	0.57***

*p ≤ 0.05; **p < 0.01; ***p < 0.001.

**FIGURE 3:**
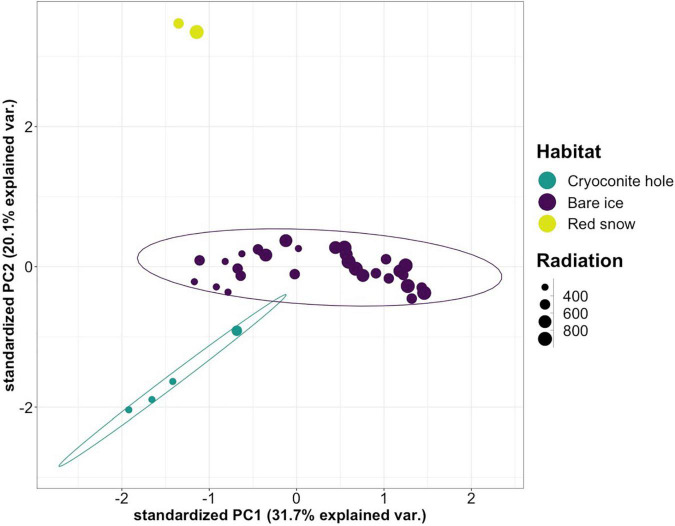
Principal component score plot for the PCA of emission rates per compound from cryoconite holes, bare ice, and red snow. Data points are sized according to the average incoming shortwave radiation in W m^–2^ at the time of sample collection. The 95% confidence ellipses are marked for cryoconite holes and bare ice, but not for red snow due to the limited number of samples (*n* = 2). For the corresponding loading plot, see Supplementary Figure 1.

Bare ice surfaces, cryoconite holes, and red snow surfaces presented distinct VOC profiles as analyzed by PCA (Figure 3). The three habitats were separated from each other along PC2, driven by compounds most characteristic of the red snow and cryoconite hole VOC profiles (Supplementary Figure 1).

**FIGURE 4:**
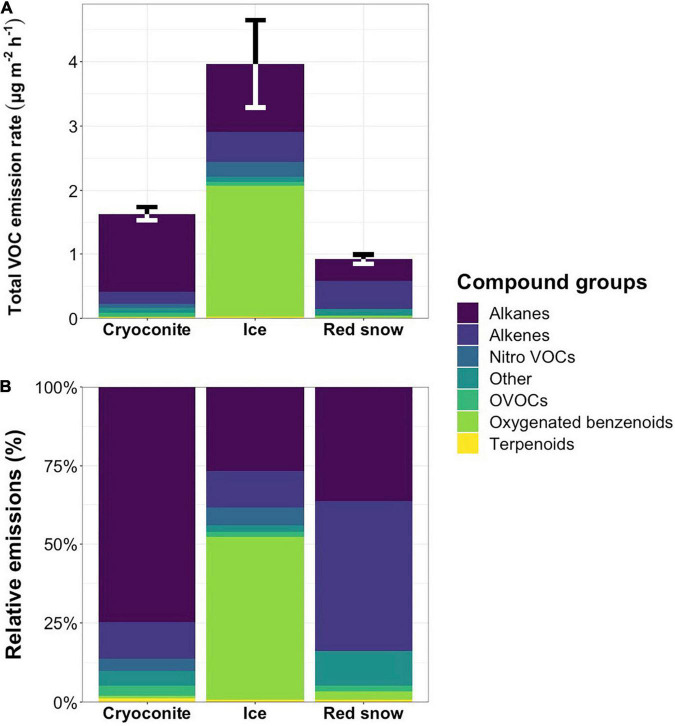
Mean total emission rates in μg m^–2^ h^–1^ **(A)** with error bars representing the standard error for total VOC emissions and mean relative emissions **(B)** per compound class from cryoconite holes, bare ice surfaces and red snow. Percentages per compound class, including standard errors, are presented in Supplementary Table 2.

Mean VOC emission rates [± standard error (SE)] were 3.97 ± 0.70 μg m^–2^ h^–1^ from bare ice surfaces (*n* = 31), 1.63 ± 0.13 μg m^–2^ h^–1^ from cryoconite holes (*n* = 4), and 0.92 ± 0.08 μg m^–2^ h^–1^ from red snow (*n* = 2), indicating that bare ice surface VOC emission rates were on average 2.4-fold and 4.3-fold higher than cryoconite hole and red snow emission rates, respectively. Oxygenated benzenoids accounted for just over half of the total emissions from bare ice surfaces (Figure 4 and Supplementary Table 2), with the three oxygenated benzenoids phenyl maleic anhydride, acetophenone and benzaldehyde accounting for 51.1 ± 11.7% of total emissions. The contributions of oxygenated benzenoids to red snow (2.8 ± 2.4%) and cryoconite hole (0.7 ± 0.2%) emissions were low. Cryoconite hole emissions were dominated by alkanes, which accounted for 74.6 ± 13.2% of emissions (Table 3 and Supplementary Table 2), while the majority of emissions from red snow consisted of alkenes (47.5 ± 6.0%, Table 4) and alkanes (36.3 ± 1.3%) (Table 3).

**TABLE 3:** Mean (ng m^–2^ h^–1^ ± SE) alkane emissions measured in three microbiological habitats on the southern Greenland ice sheet.

Compound	Red snow	Cryoconite hole	Ice surface	References
Octane	120 ± 57	n.d.	268 ± 34	Algal (Gressler et al., 2009; Sun S. M. et al., 2012) Cyanobacterial (Dembitsky et al., 1999)
Pentadecane	107 ± 37	82 ± 46	187 ± 29	Algal (Gressler et al., 2009; Yamamoto et al., 2014) Cyanobacterial (Milovanović et al., 2015)
Cyclohexane	n.d.	256 ± 53	117 ± 33	Algal (Ali, 2004) Fungal (Ezeonu et al., 1994)
Nonane	n.d.	n.d.	98 ± 20	Algal (Zuo et al., 2012b)
Methylcyclopentane	n.d.	153 ± 38	78 ± 22	Bacterial (Buszewski et al., 2008)
3-Methylpentane	n.d.	152 ± 32	69 ± 19	Algal (Garcia-Jimenez et al., 2013; Yu et al., 2019)
Tridecane	n.d.	n.d.	65 ± 12	Algal (Ali, 2004; Gressler et al., 2009)
Tetradecane	n.d.	n.d.	42 ± 8	Algal (Gressler et al., 2009) Cyanobacterial (Milovanović et al., 2015)
Undecane	n.d.	n.d.	32 ± 7	Algal (Gressler et al., 2009)
Heptadecane	38 ± 11	n.d.	24 ± 4	Algal (Gressler et al., 2009; Yamamoto et al., 2014; Berneira et al., 2021) Cyanobacterial (Milovanović et al., 2015)
1,3-Dimethylcyclopentane	n.d.	43 ± 9	13 ± 5	
2-Methyloctane	n.d.	n.d.	13 ± 3	Algal (Sun S. M. et al., 2012)
Ethylcyclopentane	n.d.	20 ± 4	9 ± 3	
Unknown alkane 1	9 ± 2	6 ± 2	9 ± 1	
3-Methylnonane	n.d.	n.d.	8 ± 2	Fungal (Ahamed and Ahring, 2011)
3-Methyltridecane	n.d.	n.d.	7 ± 1	Algal (Zhang et al., 2010)
1,2,3-Trimethylcyclopentane	n.d.	n.d.	5 ± 1	
Unknown alkane 2	n.d.	n.d.	3 ± 1	
Unknown alkane 3	n.d.	n.d.	2 ± 0	
Unknown alkane 4	3 ± 1	1 ± 0	1 ± 0	
Unknown alkane 5	n.d.	n.d.	1 ± 0	
1,1-Dimethylcyclopentane	n.d.	44 ± 11	n.d.	
1,2-Dimethylcyclopentane	n.d.	41 ± 11	n.d.	Cyanobacterial (Dembitsky et al., 1999)
1,2,4-Trimethylcyclohexane	n.d.	2 ± 1	n.d.	
1,2,4-Trimethylcyclopentane	n.d.	3 ± 1	n.d.	Fungal (Siddiquee et al., 2015)
2,3-Dimethylpentane	n.d.	37 ± 12	n.d.	
3-Methylhexane	n.d.	82 ± 28	n.d.	Algal (Fisher et al., 2020)
3,3-Dimethylpentane	n.d.	17 ± 4	n.d.	
4-Methyloctane	13 ± 5	n.d.	n.d.	
Methylcyclohexane	n.d.	233 ± 54	n.d.	
Unknown alkane 6	n.d.	34 ± 6	n.d.	
Unknown alkane 7	5 ± 1	5 ± 3	n.d.	
Unknown alkane 8	n.d.	3 ± 1	n.d.	
Unknown alkane 9	21 ± 12	n.d.	n.d.	
Unknown alkane 10	6 ± 2	n.d.	n.d.	
Unknown alkane 11	12 ± 0	n.d.	n.d.	

*n.d., not detected. When available, references are included for compounds that have previously been reported as algal, cyanobacterial, bacterial, or fungal VOCs.*

VOC emissions from bare ice surfaces did not correlate with the number (cells mL^–1^) of glacier ice algae (*A. alaskanum* and *A. nordenskiöldii*) (*r* = 0.13, *p* = 0.54, *n* = 26) or red snow algae (*r* = 0.34, *p* = 0.16, *n* = 18). The limited number of samples collected from red snow and cryoconite holes prevented a similar analysis for these habitats.

## Discussion

This study presents the first evidence for VOC emissions from snow and ice surfaces on the Greenland ice sheet inhabited by active microbial communities. The three habitats sampled in this study (bare ice, red snow, and cryoconite holes) presented different VOC profiles, with bare ice surfaces yielding the highest total emission rates (3.97 ± 0.70 μg m^–2^ h^–1^, compared to 1.63 ± 0.13 and 0.92 ± 0.08 μg m^–2^ h^–1^ from cryoconite holes and red snow, respectively). Our results show that Greenland ice sheet surfaces colonized by microbial communities are a source of VOCs. Bare ice surface VOC emission rates were approximately one-third of those detected from tundra heath (10.9 ± 2.55 and 14.62 ± 3.51 μg m^–2^ h^–1^ in unmanipulated subarctic tundra heath plots in the 2006 and 2007 growing seasons, respectively), measured using a push–pull system similar to that used in this study (Faubert et al., 2010). The bare ice extent on the Greenland ice sheet has, on average, increased by 7,158 km^2^ per year between 2000 and 2014 (Shimada et al., 2016) due to increased melt of the snowpack and is likely to expand further in a warming climate. This may lead to an overall increase in VOC emissions from Greenland ice sheet surfaces colonized by microbial communities, which present a thus far unstudied source of VOCs.

### Sampling Conditions

The average chamber headspace temperature and relative humidity did not correlate with either air temperature or air relative humidity. However, the range of relative humidity in the chambers was similar to the range of relative humidity in the ambient air, as recorded at the PROMICE weather station QAS_M, giving confidence that representative relative humidity was prevalent during sample collection. In some cases, chamber temperatures were up to five times higher than air temperatures during the time of sampling, likely due to the greenhouse effect inside the chambers. This effect cannot be mitigated in the current experimental setup. We note that the parameters recorded at the PROMICE station, namely, air temperature and air relative humidity, were measured at approximately 2.6 m above the bare ice surface. This may explain some of the temperature variation, as near-surface conditions are highly variable and are affected by near-surface meteorology and heat exchange with the glacier surface (Bravo et al., 2019). Chamber temperature and incoming shortwave radiation correlated positively (*r* = 0.63, *p* = 0.0012, *n* = 23), and the potential effect of these two parameters could not be separated in this study.

### Bare Ice Surfaces

More than 60% of the tentatively identified compounds detected in samples collected for bare ice surfaces have been reported as microbial volatiles in the literature (Tables 3–9). This suggests that the detected volatiles may be of microbial origin. Yet, no correlation was found between VOC emissions and algal counts in the top 2 cm of surface ice at the sampling sites. This lack of correlation may be explained by the fact that only the top layer of the ice surface was sampled for counts, while the bare ice surface is characterized by the presence of a porous ice weathering crust of variable depth (up to 30 cm or more) that forms due to subsurface shortwave radiation penetration, melting, and percolation. This deeper weathering crust may host microbial life (Irvine-Fynn et al., 2021) that contributes to VOC emissions but was not accounted for in the 2-cm deep surface layer used for the cell counts. Similarly, the cell counts only accounted for the presence of algae, while ice surface microbial communities are known to also include bacteria, archaea, and fungi, which likely also contributed to the measured VOC emission rates. In addition, bare ice surface microbial communities live in association with liquid water at the ice surface, indicating that volatiles are likely released into the water-phase rather than directly into the air. Microbial volatiles typically diffuse quickly through both the gas and water phases (Weisskopf et al., 2021). However, air-liquid partition coefficients are directly dependent on temperature, meaning that slower volatilization of VOCs from cold ice sheet surface waters may be anticipated. Hence, microbial volatiles may be distributed away from the source microorganisms through the hydrologically connected weathering crust (Cook et al., 2016). This is further supported by similar emission rates being detected in samples collected simultaneously (Figure 2, samples with identical incoming shortwave radiation were collected at the same time), as the distance between the sampling locations was limited to 5 m by the length of tubing on the pump system. The positive correlation between chamber temperature and incoming shortwave radiation may further interfere with a possible linear relationship between algal abundance and VOC emissions, as warming inside the chamber may lead to an increase in the volatilization of VOCs from surface water.

Kramshøj et al. (2019) suggested that the depth of the water table in permafrost soils following a thaw event influences the soil VOC emission rates, as compounds diffuse slower through water than through air. The water table in the Greenland ice sheet weathering crust is highly variable (Cooper, 2018) and data on water table depth during sample collection for this study is not available. Hence, we speculate that variations in near-surface hydrology may explain some of the variations in the measured emissions. Furthermore, a range of photochemical reactions are known to occur in snow and ice (Grannas et al., 2007). To the best of our knowledge, volatile concentrations have been measured in melted snow (Herbert et al., 2006; Kos and Ariya, 2006), but no *in situ* measurements of VOC emission rates for supraglacial microbial habitats have been reported. Ariya et al. (2011) reported (semi-)volatile concentrations ranging from 0.007 to 7.4 μg L^–1^ in melted snow samples but found no link with bacterial or fungal counts from those samples. The majority of the most abundant VOCs in our study have previously been reported as microbial VOCs, but we cannot disregard the possibility that snow or ice melt may provide non-microbial sources of volatiles, hence further confounding simple correlations of cell counts with VOC emissions. Volatile release from melting ice needs to be assessed to better quantify potential microbial contributions to bare ice surface VOC emissions.

Irradiance is one of the main drivers of ice sheet surface melt, and so any volatiles originating from the ice, as a result of melting out of recent and/or past deposition, will likely peak on sampling days with high irradiance. However, microbial contributions to the total VOC emissions from bare ice surfaces are also expected to peak at high irradiances for several reasons. First, Williamson et al. (2020) showed that glacier ice algae were more stressed when exposed to 100% ambient irradiance compared to those exposed to 50 or 0% ambient irradiance. Stress has previously been shown to induce VOC production in algae (Zuo, 2019). Second, changes in algal fatty acid biosynthesis have been shown in response to abiotic stresses (Teoh et al., 2004; Holzinger and Karsten, 2013), such as high levels of irradiance and/or freeze-thaw cycles, both of which supraglacial microbial communities are subjected to. Alkanes (Table 3) and alkenes (Table 4), which account for 38.5, 86.2, and 83.8% of emissions from bare ice, cryoconite holes, and red snow, respectively, (Figure 4 and Supplementary Table 2) are known volatile (by-)products of fatty acid biosynthesis in microorganisms (Schmidt et al., 2015; Sorigué et al., 2016). Recently, an enzyme belonging to an algae-specific subgroup of the glucose-methanol-choline oxidoreductase family, fatty acid photodecarboxylase (FAP), was identified (Sorigué et al., 2017). FAP is one of the few known enzymes that requires light for their catalytic cycle and operates independently of photosystem II (Moulin et al., 2021). Hence, both the stress caused by high levels of irradiance and the fact that a light-driven enzyme plays a role in hydrocarbon production point toward higher microbial volatile (hydrocarbon) production when irradiance is high. High light conditions have also been shown to increase emissions of isoprene and monoterpenes in cyanobacteria (Shaw et al., 2003) and algae (Meskhidze et al., 2015) and of halogenated hydrocarbons in marine algae (Bondu et al., 2008).

**TABLE 4:** Mean (ng m^–2^ h^–1^ ± SE) alkene emissions measured in three microbiological habitats on the southern Greenland ice sheet.

Compound	Red snow	Cryoconite hole	Ice surface	References
**1-Octene**	120 ± 11	n.d.	173 ± 29	Algal (Karabay-Yavasoglu et al., 2007; Zuo et al., 2015) Fungal (Matysik et al., 2008)
1-Nonene	94 ± 33	45 ± 25	72 ± 8	Fungal (Matysik et al., 2008)
1-Heptene	66 ± 5	60 ± 18	72 ± 12	Fungal (Matysik et al., 2008)
1-Hexene	38 ± 6	32 ± 8	47 ± 6	
1-Tridecene	24 ± 0	14 ± 6	33 ± 4	Algal (Kumar et al., 2011)
1-Dodecene	28 ± 8	n.d.	28 ± 5	Algal (Gressler et al., 2009; Zuo et al., 2012a)
2-Octene	16 ± 2	14 ± 4	17 ± 3	Algal (Zuo et al., 2015)
1-Tetradecene	22 ± 12	n.d.	16 ± 3	Algal (Gressler et al., 2009; Renukadevi et al., 2011)
Unknown alkene 1	12 ± 5	6 ± 3	10 ± 1	
2,4-Dimethylhept-1-ene	5 ± 2	2 ± 1	3 ± 1	Algal (Gressler et al., 2009)
1,3-Octadiene	5 ± 2	3 ± 1	3 ± 1	Algal (Sun X. et al., 2012)
1,1,3-Trimethylcyclohexane	n.d.	3 ± 1	n.d.	Algal (Borik, 2014)
3,3,5-Trimethylcyclohexene	5 ± 1	6 ± 5	n.d.	
Unknown alkene 2	n.d.	2 ± 1	n.d.	

*n.d., not detected. When available, references are included for compounds that have previously been reported as algal, cyanobacterial, bacterial, or fungal VOCs. Compounds marked in bold had exact standard matches.*

VOC production is a common defense strategy in plants (Baldwin et al., 2006), as well as in other organisms, including microorganisms (Weisskopf et al., 2021). Four oxygenated benzenoids (Table 8) were identified in bare ice surface VOC emissions, of which the three most emitted compounds (phenylmaleic anhydride, benzaldehyde, and acetophenone) have been reported to have antifungal activity (Chen et al., 2011; Boukaew et al., 2018; Calvo et al., 2020). The presence of fungi on bare ice surfaces (Perini et al., 2019) and recent evidence that parasitic fungi can penetrate the cells of snow and glacier ice algae, leading to their destruction (Fiołka et al., 2021), suggests that algal production of antifungal VOCs may have an ecological advantage. The data presented here do not allow us to identify specific sources of measured VOCs, but it is a strong first indication that supraglacial microbial habitats are a source of microbial volatile emissions that should be investigated further in more controlled and long-term field sampling campaigns, as well studies in laboratory cultures of glacier surface species, as they become available.

### Cryoconite Holes

Cryoconite holes are considered hotspots for biogeochemical cycling in supraglacial systems (Anesio et al., 2009; Cameron et al., 2012). Thus, it may initially appear surprising that the first estimates of VOC emission rates from cryoconite holes reported here are lower than those reported for bare ice surfaces. However, the microbial communities in cryoconite holes are located at the bottom of the water column, which is typically between 5 and 10 cm deep, rather than on the exposed ice surface. This means that cryoconite hole microbial communities are likely to be less impacted by abiotic stresses, such as high irradiance and rapid temperature changes. Stress is known to induce microbial VOC production in cyanobacteria (Koksharova, 2020), which are often the dominant primary producers in cryoconite hole communities. Hence, lower stress levels in cryoconite hole microbial communities compared to bare ice surface microbial communities may explain the lower emission rates detected from cryoconite holes. In addition, cryoconite hole microorganisms have been shown to recycle carbon produced by other members of the community (Sanyal et al., 2018). Volatiles present a potential carbon source (Junker and Tholl, 2013) and may thus be partially recycled within the community, rather than emitted to the atmosphere. One-third of the compounds detected in cryoconite hole emissions have previously been reported as VOCs associated with algae, bacteria, fungi, or cyanobacteria (Tables 3–6, 8, and references therein). The limited number of samples collected in this study point toward little-to-no effect of incoming shortwave radiation on cryoconite hole total VOC emissions. However, more samples collected under controlled conditions are needed to assess any possible light dependence, as well as to better constrain the estimate of VOC emission rates from cryoconite holes and their potential recycling as an *in situ* carbon source.

**TABLE 5:** Mean (ng m^–2^ h^–1^ ± SE) nitrogen-containing VOC emissions measured in three microbiological habitats on the southern Greenland ice sheet.

Compound	Red snow	Cryoconite hole	Ice surface	References
Benzonitrile	n.d.	39 ± 17	156 ± 34	
1-Cyano-1-phenylbutyl ester ethaneperoxoic acid	n.d.	4 ± 3	24 ± 5	
Benzamide	n.d.	8 ± 3	19 ± 3	
Diethyltoluamide	n.d.	4 ± 1	11 ± 3	
Hexanenitrile	n.d.	3 ± 1	6 ± 1	Fungal (Claeson et al., 2002)
Unknown nitro 1	n.d.	1 ± 0	4 ± 1	
*N*,*N*-diethylformamide	n.d.	4 ± 3	1 ± 0	

*n.d., not detected; nitro, nitrogen-containing VOC. When available, references are included for compounds that have previously been reported as algal, cyanobacterial, bacterial, or fungal VOCs.*

**TABLE 6:** Mean (ng m^–2^ h^–1^ ± SE) other VOC emissions measured in three microbiological habitats on the southern Greenland ice sheet.

Compound	Red snow	Cryoconite hole	Ice surface	References
Unknown other 1	37 ± 29	11 ± 1	26 ± 4	
α-Methylstyrene	21 ± 6	13 ± 6	21 ± 2	Cyanobacterial (Ye et al., 2018)
Tert-butyl benzene	7 ± 2	8 ± 5	12 ± 2	
Unknown other 2	n.d.	29 ± 7	11 ± 3	
Unknown other 3	11 ± 4	4 ± 1	6 ± 1	
1,2-Dichlorobenzene	5 ± 1	1 ± 0	2 ± 2	
Unknown other 4	4 ± 0	n.d.	2 ± 0	
3-Methylfuran	n.d.	29 ± 5	n.d.	Algal (Fisher et al., 2020) Fungal (Borjesson et al., 1992)
Unknown halogen 1	2 ± 2	n.d.	n.d.	
Unknown other 5	4 ± 0	n.d.	n.d.	
Unknown other 6	6 ± 3	n.d.	n.d.	
Unknown other 7	4 ± 1	n.d.	n.d.	
Unknown other 8	n.d.	3 ± 1	n.d.	
Unknown sulfo 1	n.d.	7 ± 1	n.d.	

*n.d., not detected. When available, references are included for compounds that have previously been reported as algal, cyanobacterial, bacterial, or fungal VOCs.*

**TABLE 7:** Mean (ng m^–2^ h^–1^ ± SE) OVOC emissions measured in three microbiological habitats on the southern Greenland ice sheet.

Compound	Red snow	Cryoconite hole	Ice surface	References
Unknown OVOC 1	n.d.	11 ± 7	42 ± 8	
1H-indene-1,3(2h)-dione	n.d.	2 ± 1	12 ± 3	
2-Ethylhexyl ester formic acid	8 ± 4	5 ± 3	8 ± 1	
Unknown aldehyde 1	n.d.	2 ± 1	4 ± 1	
Cyclohexanepropanol	n.d.	6 ± 2	n.d.	
1-(1-Cyclohexen-1-yl)-ethanone	3 ± 1	n.d.	n.d.	
Unknown OVOC 2	3 ± 3	n.d.	n.d.	

*n.d., not detected; OVOC, oxygen-containing VOC. When available, references are included for compounds that have previously been reported as algal, cyanobacterial, bacterial, or fungal VOCs.*

**TABLE 8:** Mean (ng m^–2^ h^–1^ ± SE) oxygenated benzenoid emissions measured in three microbiological habitats on the southern Greenland ice sheet.

Compound	Red snow	Cryoconite hole	Ice surface	References
Phenylmaleic anhydride	n.d.	n.d.	1,049 ± 227	
**Benzaldehyde**	n.d.	n.d.	754 ± 187	Algal (Gressler et al., 2009; Sun S. M. et al., 2012) Bacterial (Zou et al., 2007; Calvo et al., 2020)
**Acetophenone**	n.d.	n.d.	223 ± 53	Algal (Kamenarska et al., 2000) Fungal (Kim et al., 2018)
Unknown obz 1	n.d.	2 ± 1	10 ± 2	
Unknown obz 2	4 ± 3	3 ± 2	6 ± 1	
4-(1,1-Dimethylpropyl)-phenol	15 ± 13	2 ± 1	2 ± 1	Fungal (Bhardwaj et al., 2017)
Unknown obz 3	7 ± 6	1 ± 0	2 ± 0	
2,4-Di-tert-butylphenol	n.d.	4 ± 1	n.d.	Algal (Gressler et al., 2009; Vahdati et al., 2022) Bacterial (Zhao et al., 2020) Fungal (Zhao et al., 2020)

*n.d., not detected; obz, oxygenated benzenoid. When available, references are included for compounds that have previously been reported as algal, cyanobacterial, bacterial, or fungal VOCs. Compounds marked in bold had exact standard matches.*

**TABLE 9:** Mean (ng m^–2^ h^–1^ ± SE) terpenoid emissions measured in three microbiological habitats on the southern Greenland ice sheet.

Compound	Red snow	Cryoconite hole	Ice surface	References
Unknown monoterpene 1	n.d.	13 ± 2	24 ± 4	
**Linalool**	n.d.	1 ± 0	2 ± 0	
Unknown oxygenated sesquiterpene 1	4 ± 2	5 ± 3	2 ± 0	
*Cis*-α-bergamotene	1 ± 0	n.d.	n.d.	
Unknown sesquiterpene 2	1 ± 0	n.d.	n.d.	
Unknown sesquiterpene 3	1 ± 0	n.d.	n.d.	

*n.d., not detected. When available, references are included for compounds that have previously been reported as algal, cyanobacterial, bacterial, or fungal VOCs. Compounds marked in bold had exact standard matches.*

### Red Snow

Red snow patches near the 2020 basecamp were dominated by uncultured Chlamydomonadaceae (18S sequencing data, unpublished). One-third of compounds detected in red snow VOC emissions have been reported as microbial volatiles (Tables 3, 4, 6, 8, and references therein). Of these, 1-octene, 1-dodecene, and 1-tetradecene have been reported as volatiles from *C. reinhardtii* (Renukadevi et al., 2011; Zuo et al., 2015), a well-studied model organism that also belongs to the Chlamydomonadaceae family. We noted that 1-octene and 1-dodecene were detected in normal *C. reinhardtii* cells, as well as in *C. reinhardtii* cells subjected to programmed cell death (Zuo et al., 2015). Programmed cell death is hypothesized to be a pro-survival mechanism at the population level (de Carpentier et al., 2019) and may be an interesting future area of study in terms of controls on snow algal communities.

## Conclusion

This study is the first to report VOC profiles and emission rates from Greenland ice sheet surfaces colonized by active microbial snow, ice, and cryoconite hole communities. Bare ice surfaces present the largest source of VOCs, with emission rates (3.97 ± 0.70 μg m^–2^ h^–1^) comparable to roughly one-third of those detected from tundra heath. This is especially interesting given that Greenland ice sheet bare ice surfaces present a source of VOCs in an area that has few other local VOC sources and that the Greenland bare ice extent is increasing in our planet’s warming climate. No direct correlation with ice surface algal counts was found, but the data presented here provide strong evidence that at least some of the detected VOCs are of microbial origin. A weak positive correlation between incoming shortwave radiation and VOC emission rates from bare ice surfaces was found, possibly indicating increased VOC emissions by supraglacial microorganisms in response to stress induced by high solar irradiance. Given that incoming shortwave radiation is correlated with chamber temperature, a microbial response to temperature increase, or increased volatilization from the liquid phase due to a local rise in temperature, may also contribute to higher VOC emission rates. Some of the main volatiles detected in bare ice surface emissions are known to have antifungal activity, pointing toward VOC emissions as a possible defense strategy against fungal infections in bare ice microbial communities. First estimates of VOC emissions from cryoconite holes (1.63 ± 0.13 μg m^–2^ h^–1^) and red snow (0.92 ± 0.08 μg m^–2^ h^–1^) are presented, suggesting that these habitats are also contributors to Greenland ice sheet VOC emissions. Further work is needed to identify constraints on and sources of supraglacial VOC emissions, focusing on both measurements in controlled and isolated systems, such as pure cultures or incubation experiments, and field measurements at different sites, under more controlled conditions and throughout the ablation season.

## Data Availability Statement

The raw data supporting the conclusions of this article will be made available by the authors, without undue reservation.

## Author Contributions

All authors contributed with ideas to the experimental design. ELD collected all samples, carried out data analyses, prepared all figures, and wrote the first manuscript draft. CLD-M performed all GC–MS and PARADISe analyses. All authors contributed to the writing phase with discussion and revisions.

## Conflict of Interest

The authors declare that the research was conducted in the absence of any commercial or financial relationships that could be construed as a potential conflict of interest.

## Publisher’s Note

All claims expressed in this article are solely those of the authors and do not necessarily represent those of their affiliated organizations, or those of the publisher, the editors and the reviewers. Any product that may be evaluated in this article, or claim that may be made by its manufacturer, is not guaranteed or endorsed by the publisher.

## Footnotes

1
https://www.deeppurple-ercsyg.eu/home

2
https://www.promice.org

## Supplementary Material

Supplementary Figure 1 Loading plot for the PCA of emission rates per compound from cryoconite holes, red snow, and bare ice surfaces.

Supplementary Table 1 Pure standards and the compound classes they were assigned to for the quantification of VOC emission rates. If no pure standard was available, the standard most closely resembling the structure of the tentatively identified compound was assigned for quantification purposes.

Supplementary Table 2 Mean proportional contributions (± SE) of each compound group to the total VOC emissions measured from bare ice surfaces, cryoconite holes, and red snow surfaces.
