# Supplementary figures and images for: Greenland Ice Sheet Surfaces Colonized by Microbial Communities Emit Volatile Organic Compounds
Source: Front Microbiol. 2022 Jun 7;13:886293. doi: 10.3389/fmicb.2022.886293 (PMC9211068; doi:10.3389/fmicb.2022.886293)

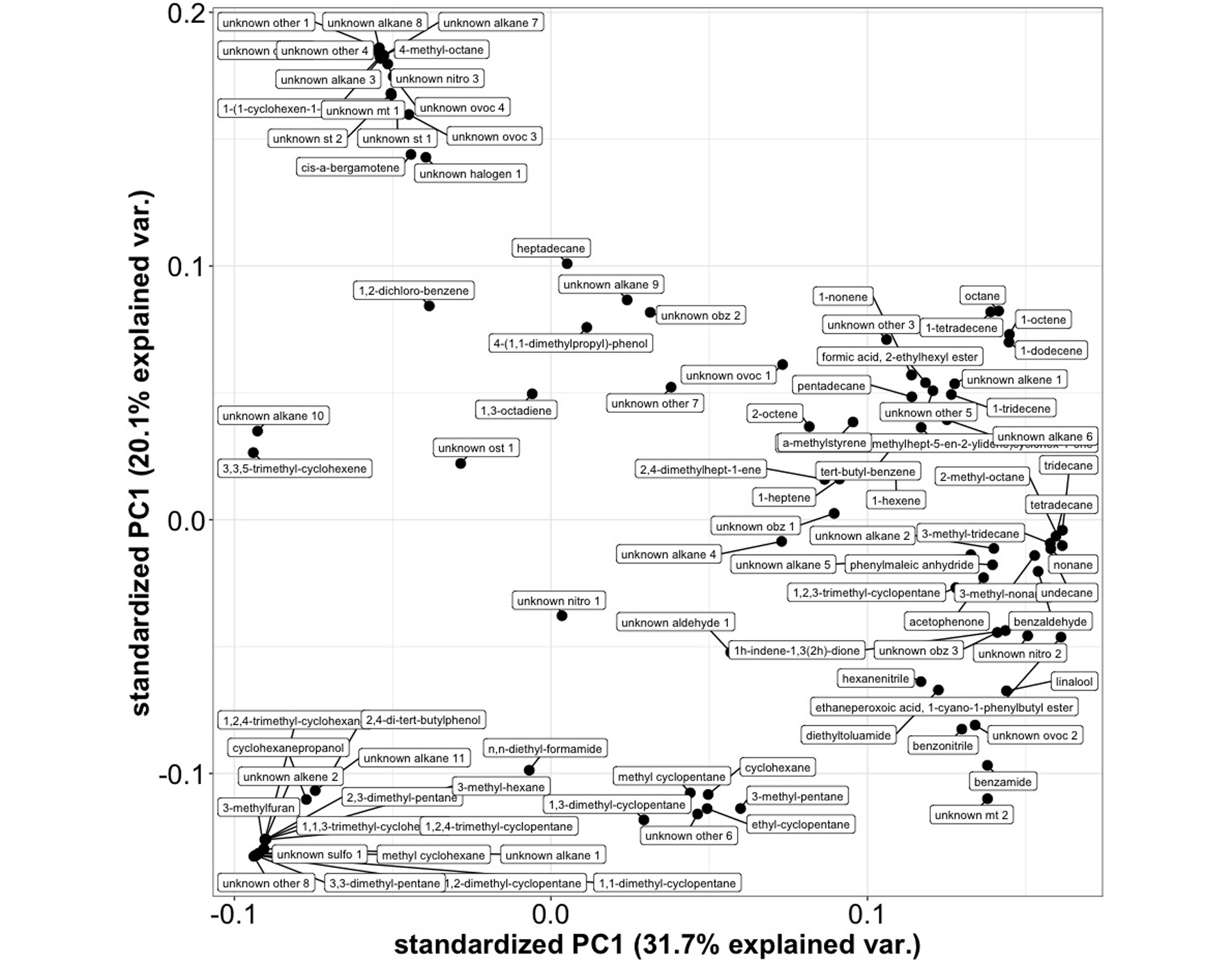

Supplement: Supplementary Figure 1 — Loading plot for the PCA of emission rates per compound from cryoconite holes, red snow, and bare ice surfaces. [file Image_1.JPEG]
