# Supplementary material for: Greenland Ice Sheet Surfaces Colonized by Microbial Communities Emit Volatile Organic Compounds
Source: Front Microbiol. 2022 Jun 7;13:886293. doi: 10.3389/fmicb.2022.886293 (PMC9211068; doi:10.3389/fmicb.2022.886293)
Supplement: Supplementary Table 1 — Pure standards and the compound classes they were assigned to for the quantification of VOC emission rates. If no pure standard was available, the standard most closely resembling the structure of the tentatively identified compound was assigned for quantification purposes. [file Table_1.DOCX]

**Supplementary Table 1 |** Pure standards and the compound classes they were assigned to for the quantification of VOC emission rates. If no pure standard was available, the standard most closely resembling the structure of the tentatively identified compound was assigned for quantification purposes.

| **Standard** | **Retention time**  **(min)** | **Assignment** |
| --- | --- | --- |
| Isoprene | 4.160 | Isoprene <C6 alkenes |
| 2-Butanone | 5.288 | <C8 ketones |
| 2-Methylfuran | 5.434 | Furans (single O) |
| Toluene | 9.874 | Benzenoids (simple), other (-Si, -S, -F/Cl/Br) |
| 1-Octene | 10.598 | <C12 alkanes, simple alkenes |
| Hexanal | 10.910 | <C8 aldehydes |
| 2-Furaldehyde | 12.104 | Oxygenated furans |
| trans-2-Hexen-1-al | 12.804 | Oxygenated alkenes |
| cis-3-Hexen-1-ol | 12.877 | <C8 alcohols |
| p-Xylene | 13.445 |  |
| o-Xylene | 14.356 |  |
| a-Pinene | 15.841 | Monoterpenes (C10H16, C10H14) |
| Camphene | 16.403 | Cyclic alkanes, >C12 alkanes |
| Benzaldehyde | 16.754 | Oxygenated benzenoids (single O) |
| 1-Octen-3-ol | 17.194 | C8 alcohols |
| B-Pinene | 17.387 |  |
| Myrcene | 17.659 |  |
| Octanal | 18.056 | C8 aldehydes, C8 ketones |
| cis-3-Hexenyl acetate | 18.126 | ≤ C9 esters, ≤C9 acids, ≤C9 ethers, ≤C9 OVOCs |
| a-Phellandrene | 18.261 |  |
| 3-Carene | 18.478 |  |
| p-Cymene | 18.930 |  |
| Limonene | 19.082 |  |
| Benzyl alcohol | 19.156 |  |
| Eucalyptol | 19.217 | Oxygenated monoterpenes (C10H18O, C10H16O) |
| Ocimene | 19.577 | Multiple (di-, tri-) alkenes, >12C alkenes |
| y-Terpinene | 20.057 |  |
| Acetophenone | 20.350 |  |
| Terpinolene | 21.056 | Cyclic alkenes |
| Linalool | 21.258 | >C8 alcohols |
| Nonanal | 21.423 | >C8 aldehydes, >C8 ketones |
| (+)-Camphor | 23.027 |  |
| (+)-Borneol | 23.638 |  |
| cis-3-Hexenyl butyrate | 23.882 | >C9 esters, >C9 acids, >C9 ethers, >C9 OVOCs |
| Methyl salicylate | 24.457 | Highly oxygenated benzenoids |
| Bornyl acetate | 27.112 |  |
| Indole | 27.329 | Nitrogen-based other |
| B-Caryophyllene | 31.038 | Sesquiterpenes (C15H24, C15H22) |
| Aromandendrene | 31.545 |  |
| a-Humulene | 31.924 |  |
| Nerolidol | 34.173 | Oxygenated sesquiterpenes (C15H26O, C15H24O) |
