# Supplementary material for: Greenland Ice Sheet Surfaces Colonized by Microbial Communities Emit Volatile Organic Compounds
Source: Front Microbiol. 2022 Jun 7;13:886293. doi: 10.3389/fmicb.2022.886293 (PMC9211068; doi:10.3389/fmicb.2022.886293)
Supplement: Supplementary Table 2 — Mean proportional contributions (± SE) of each compound group to the total VOC emissions measured from bare ice surfaces, cryoconite holes, and red snow surfaces. [file Table_2.DOCX]

**Supplementary Table 2 |** Mean proportional contributions (± standard error) of each compound group to the total VOC emissions measured from bare ice surfaces, cryoconite holes and red snow surfaces.

| **Name** | **Bare ice (%)** | **Cryoconite hole (%)** | **Red snow (%)** |
| --- | --- | --- | --- |
| Alkanes | 26.5 ± 3.9 | 74.6 ± 13.2 | 36.3 ± 1.3 |
| Alkenes | 12.0 ± 1.6 | 11.6 ± 3.75 | 47.5 ± 6.0 |
| Terpenoids | 0.7 ± 0.1 | 1.2 ± 0.2 | 0.7 ± 0.4 |
| Oxygenated benzenoids | 51.6 ± 11.8 | 0.7 ± 0.2 | 2.8 ± 2.4 |
| OVOCs | 1.6 ± 0.3 | 3.4 ± 0.7 | 1.7 ± 0.3 |
| Nitro VOCs | 5.6 ± 1.2 | 3.8 ± 1.4 | 0.0 ± 0.0 |
| Other | 2.0 ± 0.2 | 4.7 ± 0.4 | 11.1 ± 2.5 |
